# Facile Synthesis of Highly Crystalline and Large Areal Hexagonal Boron Nitride from Borazine Oligomers

**DOI:** 10.1038/srep40260

**Published:** 2017-01-11

**Authors:** Sungchan Park, Tae Hoon Seo, Hyunjin Cho, Kyung Hyun Min, Dong Su Lee, Dong-Il Won, Sang Ook Kang, Myung Jong Kim

**Affiliations:** 1 Applied Quantum Composites Research Center, Korea Institute of Science and Technology, Chudong-ro 92, Bongdong-eup, Wanju-gun, Jeollabuk-do, 565-905, Republic of Korea; 2 Department of Advanced Materials Chemistry, Korea University, 2511 Sejong-ro, Sejong, 30019 Republic of Korea

## Abstract

A novel and facile synthetic method for h-BN films from borazine oligomer (B_3_N_3_H_4_)_x_ precursors has been developed. This method only includes spin-coating of borazine oligomer onto nickel catalysts and a subsequent annealing step. Large areal and highly crystalline h-BN films were obtained. The stoichiometric B/N ratio of borazine oligomer precursor was preserved in the final h-BN product such that it was close to 1 as revealed by XPS. Catalytic effect of nickel for h-BN formation was clearly demonstrated by lowering crystallization temperature compared to the growth condition in the absence of catalyst. The graphene field effect transistor (GFET) characterization has proved the high quality synthesis of h-BN films, showing the shift of neutrality point and the increase of the mobility. This method can also provide functional h-BN coating on various surfaces by annealing Ni-coated borazine oligomer films and subsequent removal of Ni catalyst.

A hexagonal boron nitride (h-BN) is an analogue of graphite, stacked graphene layers where one carbon atom has sp^2^-hybridized bonding with three other carbon atoms, forming honeycomb structures. In the h-BN, boron atom has sp^2^-hybridized bonding with three nitrogen atoms, and so does nitrogen atom with three boron atoms in honeycomb structure^1^,^2^,^3^. Due to strong sp^2^ bonding, the h-BN has remarkable mechanical properties such as high tensile strength and modulus similar to graphite^4^. Difference in electronegativity between boron and nitrogen give rise to permanent dipole such that the h-BN is electrically insulating while graphite is conducting^5^. The h-BN also has a variety of useful properties such as low dielectric constant^6^, high thermal conductivity^7^, thermal and chemical stability^8^, superior lubrication^9^, neutron shielding^10^ and piezoelectricity^11^. Recently, h-BN films, often dubbed as “white graphene” have been given a considerable attention since graphene was discovered in 2004^12^. Not only structural similarity but also complementary properties triggered intensive research on the h-BN film. In particular, the h-BN substrate improved the carrier mobility of graphene due to atomically flat surface free of charge traps and dangling bonds^13^,^14^.

The h-BN film has been synthesized through various methods such as mechanical exfoliation^15^, chemical exfoliation^16^, sputtering^17^ and chemical vapor deposition (CVD)^18^,^19^,^20^,^21^. Among those methods, CVD is the most effective method due to its convenience and the ability to produce large areal h-BN films with high quality. However, the CVD method for the h-BN has a couple of drawbacks such as difficulty in handling explosive or toxic gas and high cost instrumentations. The precursor for the h-BN CVD synthesis have been also limited to borazine (B_3_N_3_H_6_)^18^, decaborane/ammonia^19^, B-trichloroborazine (ClBNH)_3_^ ^20, and ammonia borane (NH_3_BH_3_)^21^.

In this report, we firstly used the borazine oligomer as a precursor for the synthesis of highly crystalline h-BN films on metal catalyst. The borazine oligomer has advantage over others in that it can be easily coated on a variety of catalytic substrates with controlled thickness, and it also does not evaporate from the substrate in contrast with ammonia borane. Previously, the synthesis of h-BN thin films from polyborazylene or borazine oligomer at ~1000 °C has been reported^22^,^23^,^24^, but they were usually boron rich and have poor crystalline structure due to the absence of catalyst. By associating catalyst below or on top of borazine oligomer film as illustrated in Fig. 1, we were able to acquire highly crystalline and large areal h-BN films with the stoichiometric B/N ratio close to 1 at ~1000 °C. The quality of the h-BN film was tested by the transfer characteristics measured for the graphene field effect transistor (GFET), where the h-BN film is inserted as a support layer between the graphene channel and the thermally-grown SiO_2_ dielectric layer. The field effect mobility of the GFET with the h-BN layer is higher than that of the device without an h-BN layer. This implies that our h-BN films provide better substrate for device performance than the SiO_2_ due to better flatness and less embedded charges. Since the catalyst deposited on top of borazine oligomer films can be easily removed, we can coat various surfaces with high quality h-BN films for the desired purpose. The method developed here is facile and cost-effective to synthesize h-BN films for various applications.

## Results

**Figure 2:**
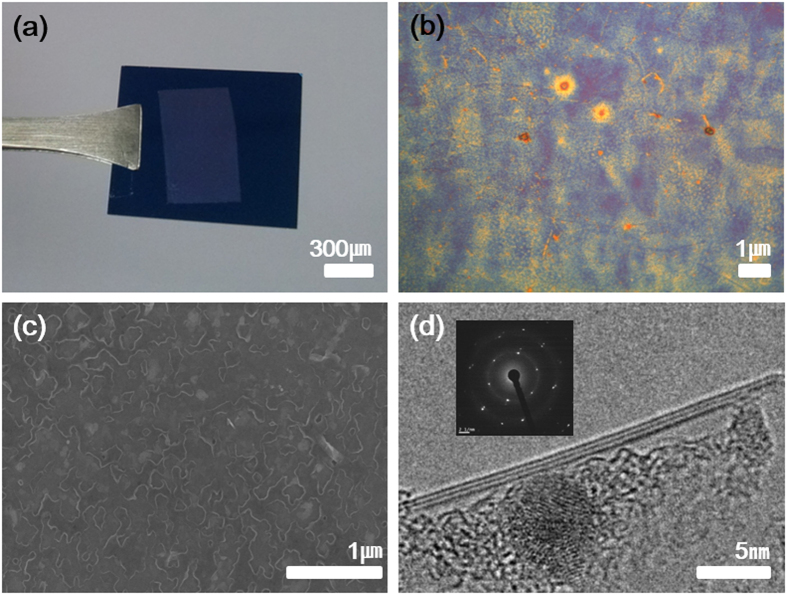
(**a**) Photographic image, (**b**) microscope image (**c**) Scanning electron microscopy (SEM) image, and (**d**) Transmission electron microscopy (TEM) image and electron diffraction (inset) of the h-BN film synthesized on a Ni foil. (**a**), (**b**) and (**c**) are taken from the transferred h-BN on the SiO_2_/Si substrate.

Figure 2 shows from macro to microscopic characterization of the h-BN film synthesized on a Ni foil. The photographic image (Fig. 2(a)) of the transferred h-BN film on a SiO_2_/Si substrate displayed that entire h-BN film was transferred on the SiO_2_/Si substrate. The whitish appearance of the sample is originated from the large bandgap (~6 eV) and defect induced scattering in the h-BN. The surface morphology of the h-BN film formed on the SiO_2_/Si substrate was characterized by optical microscope and SEM as shown in Fig. 2(b) and (c). Entire area within the edge of the h-BN film was fully occupied without voids, but surface roughness was not as good as the h-BN film synthesized by CVD. The roughness was estimated by atomic force microscopy measurements to be around 5 to 10 nm in root mean square (Fig. S4, Supporting Information). In order to characterize crystallinity and nanostructure, TEM and selected area electron diffraction (SAED) data were collected from the h-BN film transferred onto perforated lacey carbon grids. Figure 2(d) is a cross-sectional TEM image of the h-BN film, which showed clearly distinguished h-BN graphitic layers. The number of h-BN layer was 3~4 on average, as one of the examples is shown in Fig. 2(d). The h-BN film with maximum ~15 layers was sparsely observed as shown in Fig. 4(a). The ripples of the TEM image (Fig. 2(d)) can be amorphous BN, which was not fully crystallized during the annealing step. Weak circular patterns in electron diffraction (Fig. 2(d) inset) proves that the top surface of the h-BN can be covered by a thin layer of amorphous BN because Ni catalyst could not affect the top surface due to relatively long distance. The particle shown in the TEM image (Fig. 2(d)) is a Fe nanoparticle. When the h-BN film grown on a Ni foil was transferred to arbitrary substrate including a TEM grid, FeCl_3_ solution was used to etch the Ni foil. FeCl_3_ might form Fe nanoparticles when the sample was dried or when it was under e-beam irradiation during SEM or TEM characterization. EDS (Energy Dispersive Spectroscopy, Fig. S5) in SEM equipment proved the existence of Fe atoms in the h-BN film, which was transferred on a SiO_2_/Si substrate. The inset in Fig. 2(d) is the SAED pattern collected from the h-BN film, and it clearly showed hexagonal lattice structure, representing the Bernal or turbostratic stacking configuration^25^. A high resolution TEM image (Fig. S1(b), Supporting Information) also proves that the h-BN film is highly crystalline by showing triangle or honeycomb pattern depending on stacking order.

**Figure 3:**
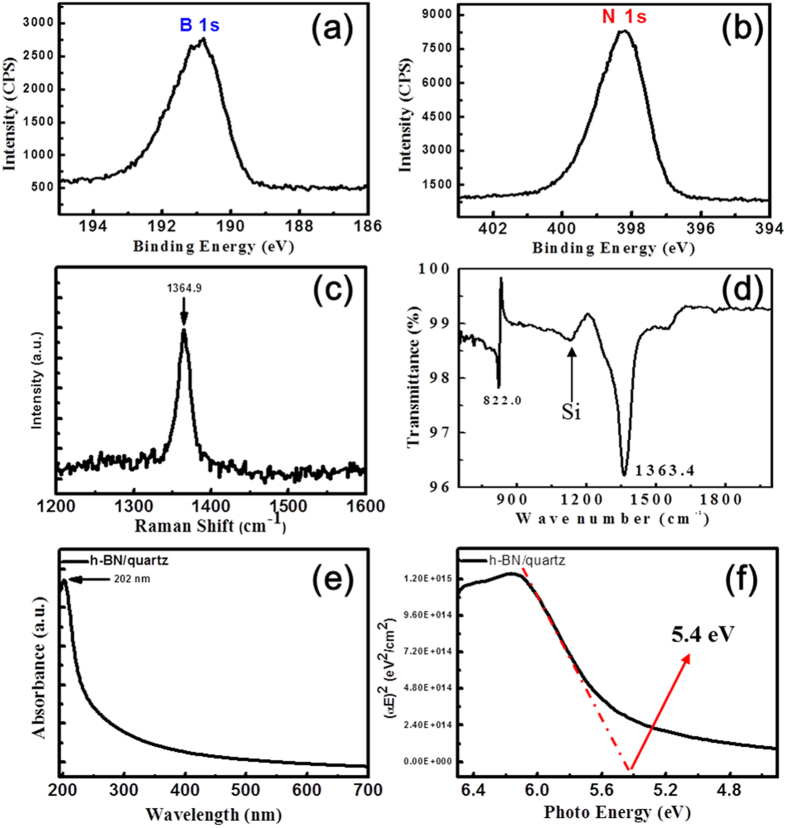
(**a**,**b**) XPS spectra, (**c**) Raman spectrum, and (**d**) FTIR spectrum of the h-BN film transferred onto a SiO_2_/Si substrate. (**e**) Absorption spectrum of h-BN formed on quartz and (**f**) graphical representation of the bandgap calculation.

XPS analysis was performed to evaluate the chemical composition and the elemental stoichiometry the h-BN film. Figure 3(a) and (b) showed the binding energies of boron 1 s and nitrogen 1 s at 190.55 eV and 399.1 eV, respectively. The results were consistent with the reported binding energy of the h-BN^26^,^27^. The stoichiometric B/N ratio was determined as 1.10. The XPS data here gives evidence that h-BN was formed with reasonable stoichiometry close to 1 to 1 ratio. This is significantly different from the previous studies^22^,^23^,^24^ that showed ~1.4 B/N ratio at 900–1000 °C without using catalysts.

The Raman spectrum was also collected from the transferred h-BN film on a SiO_2_/Si substrate as presented in Fig. 3c. The peak at 1364.9 cm^−1^ indicated the lattice vibration mode of the h-BN in-plane motion (i.e. E_2g_ mode). The peak at ~1366 cm^−1^ corresponds to the h-BN, while the cubic structure is displayed at 1300 cm^−1^ (longitudinal optical (LO) vibration mode) and at 1065 cm^−1^ (transverse optical (TO) vibration mode). The vibration mode of cubic boron nitride (c-BN) was not observed in our sample. Regarding the number of the layers of h-BN, the E_2g_ mode of a bulk h-BN is positioned around 1366 cm^−1^. When it gets thinner, the E_2g_ mode is positioned at 1368~1370 cm^−1^ for the mono-layer and at 1364~1367 cm^−1^ for the 2~5 layers^28^,^29^. In our observation, the h-BN peak is positioned at 1364.9 cm^−1^, and thus this suggests that the h-BN film 2~5 layers. This result is consistent with the acquired TEM images of the sample (Fig. 2(d)). The FWHM of E_2g_ mode is an important parameter in the measurement of crystal size because it is related to the life time of phonon. The FWHM value of the h-BN film was 18 cm^−1^, which is close to that of the h-BN film synthesized by CVD^30^. Figure 3(d) showed FT-IR spectrum with two strong absorption peaks at 818.7 cm^−1^ and 1368.9 cm^−1^, which corresponds to the LO vibration of out-of-plane B-N-B bending and TO vibration of in-plane B-N stretching, respectively. These strong absorption peaks are in good agreement with the value reported on the h-BN film synthesized by CVD^31^. Similar to the Raman active vibration mode, these modes were also useful to characterize the h-BN structure. Both the TO vibration mode of c-BN at 1065 cm^−1^ and the C-C vibration mode at 1950 cm^−1^ were not observed. To confirm the optical band gap of the h-BN film, we acquired the UV-visible absorption spectrum of the h-BN film transferred onto the quartz. From the absorption spectrum, the optical band gap can be calculated via the Kubelka Munk formula. The equation is given as α = A(E − E_g_)^1/2^/E^ ^26, where α is the absorption coefficient, A is a constant, E is the photon energy and E_g_ is the bandgap energy. Figure 3(f) shows the plotting (αE)^2^ as a function of photo energy. The optical bandgap can be estimated as approximately 5.4 eV. The optical band gap of h-BN was previously reported from 5.2 eV to 6.0 eV depending on the strain and surface roughness.

As a controlled experiment to elucidate catalyst effects, the synthesis of h-BN film was attempted on a Si substrate without catalyst at the same temperature (1026 °C). Raman and XRD spectra were collected from the h-BN films with and without catalyst as presented in Fig. S2(a) and (b) of supporting information. The E_2g_ mode, characteristics of crystalline h-BN, was not observed from the h-BN film synthesized without catalyst in the Raman spectra (Fig. S2(a)). Also, the h-BN peaks at 26.4° and 54.8° corresponding to reflection peaks of (002) and (004) planes was weaker and up-shifted in the h-BN film without catalyst, as shown in the XRD data (Fig. S2(b)). Since the high crystalline h-BN structure formed exclusively on Ni catalyst foils, we can conclude that Ni acted as a catalyst for the formation of the h-BN from borazine oligomers. The nickel catalyst appears to promote the dehydrogenation reaction during two-dimensional phase conversion to form h-BN.

## Discussion

**Figure 4:**
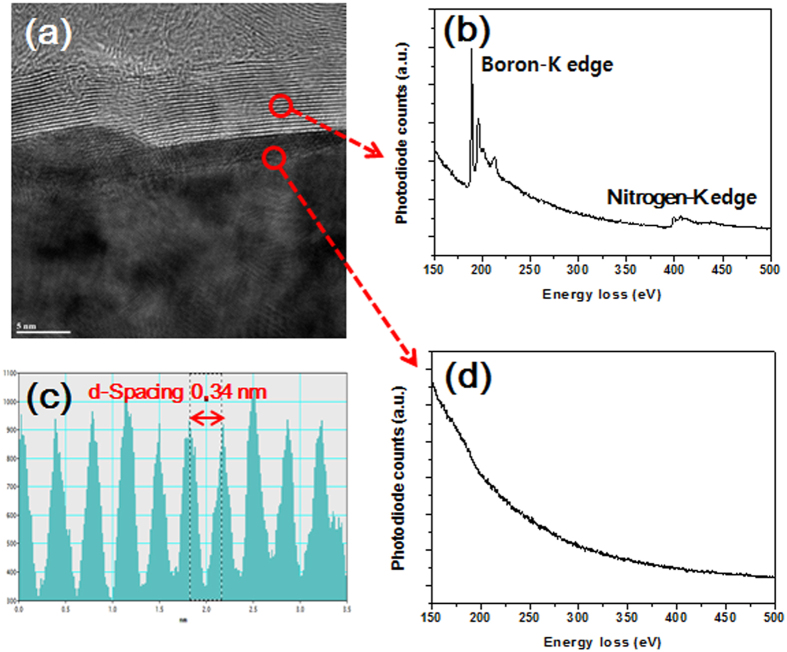
(**a**) Cross-sectional TEM image, (**b**,**d**) related EELS analysis, and (**c**) analysis for the layer spacing.

The mechanism of the h-BN formation on Ni catalyst from borazine oligomers can be understood similar to the previous report on h-BN formation in the absence of catalyst. At the initial period of sample heating, borazine oligomers begin to form 2-dimensional structures via dehydro-crosslinking between the oligomers and they become fully crystallized into h-BN structure at 1026 °C for 60 minutes’ duration. Since Ni is known as an efficient catalyst for the dehydrogenation of ammonia borane to produce borazine, it can also catalyze the dehydro-crosslinking reaction between borazine oligomers. Crystallization of the 2-dimensional structures into h-BN on Ni catalyst might not be same to the metal induced crystallization (MIC) of amorphous carbon into graphite or graphene, where carbon soluble metal catalysts such as Ni and Co were used. The dissolution-precipitation mechanism that governed the crystallization of amorphous carbon cannot be adapted to that of polyborazylene because Ni has 0.3 at.% solubility of boron at 1085 °C and almost zero solubility of nitrogen above 445 °C^32^. Though boron can be dissolved into Ni catalyst considering its solubility, boron atoms within the cross-linked 2-dimensional BN structures formed during initial growth cannot be dissolved in Ni catalyst due to the strong bonds between boron and nitrogen. Thus, crystalline h-BN layers might be formed on Ni catalyst surface from the bottom to the top layer. Figure 4 revealed the bonding structure and elemental components of the synthesized h-BN film by electron energy loss spectroscopy (EELS) analysis. The elemental composition such as boron and nitrogen was confirmed by observing the K-shell ionization edges of boron at 188 eV and nitrogen at 401 eV, which indicated sp^2^ hybridization bonding of h-BN^33^. As Fig. 4 demonstrates, boron and nitrogen K-edges identified from the h-BN layers (Fig. 4(b)) did not appear right below the h-BN layers irrespective of cooling rate (Fig. 4(d)). This implies that the crystalline h-BN film was formed not by the dissolution-precipitation but by the surface catalysis of the Ni films.

h-BN film synthesis under a nickel catalyst layer was also attempted in order to coat h-BN layers directly on a variety of substrates without a transfer process. After the h-BN synthesis, a top nickel catalyst layer was etched by using Iron (III) chloride (FeCl_3_) etchant. Then, we obtained the Raman and XRD spectra of the h-BN films so as to compare structural characteristics of the h-BN films before and after etching as shown in Fig. S3(a) and (b) of supporting information. In Raman spectrum, the major peak associated with E_2g_ of h-BN is observed at 1366 cm^−1^~1367 cm^−1^. The fact that the E_2g_ mode peak existed after etching proves its chemical stability. In addition, the prominent peaks implicated in h-BN structure and reflection peak of (002) and (004) were manifested at 26.4° and 54.8°, respectively, as shown in XRD spectrum of Fig. S3(b)^34^. After etching, on the other hand, the two peaks (44.4° and 51.9°) corresponded to the (111) and (200) planes of the nickel disappeared in the XRD spectrum, implying the removal of nickel catalyst^35^. Thus, both Raman and XRD data support that the h-BN films are intact after etching with Iron (III) chloride (FeCl_3_) solution. This method can provide the h-BN functional coating on various substrates for the purpose of insulation, thermal management, and chemical or thermal protection.

**Figure 5:**
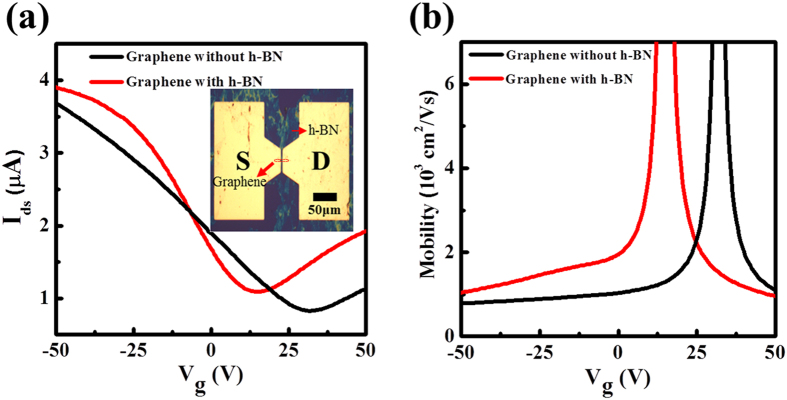
(**a**) The source-drain current of GFETs fabricated on a SiO_2_ substrate and on a h-BN/SiO_2_ substrate as a function of back gate bias at room temperature with a V_ds_ = 5 mV. (**b**) Carrier mobility with back gate bias for GFETs with and without h-BN. (Inset) Optical image of the fabricated GFETs with h-BN on SiO_2_/Si substrate.

For the applications of the synthesized h-BN, the transfer characteristics of the graphene transistor where the h-BN film is inserted as a support layer between the graphene channel and the SiO_2_ gate dielectric layer has been measured. It has already been known that the flakes exfoliated from single crystalline h-BN improves the GFET performance due to their atomic flatness, less embedded charge impurities and higher optical phonon energies compare to SiO_2_^36^,^37^,^38^. The fabricated GFET structure is shown in the inset of Fig. 5. For channel material graphene grown by CVD method was transferred on top of our synthesized h-BN film. A GFET without the h-BN layer was also fabricated for comparison. More details for fabrication are deferred to the *Method*. Figure 5(a) shows the drain-source current as a function of backgate voltage (*V*_g_) for the devices. For both transfer curves charge neutrality point (*V*_N_) was observed at positive gate voltage showing typical *p*-type behaviour. The charge neutrality points for the devices with and without the h-BN film appear at 10.5 V and 32 V, respectively. The doping level can be obtained by *n*_doping_ = α*V*_N_. where *n* is the charge carrier density and α ~ 7.2 × 10^10^ cm^−2^ V^−1^ (for a 300 nm-thick SiO_2_) is the gate efficiency factor. The hole doping concentration for the GFETs with and without the h-BN film is ~7.6 × 10^11^ and ~2.3 × 10^12^ cm^−2^, respectively. The GFET with the h-BN support shows less p-type doping concentration, which is consistent with the previous reports with the h-BN support. The field-effect mobility obtained from the transfer characteristics by μ = *G·(l/w*)·[1/(eα*V*_g_)], where *G* is the conductance and *l (w*) is the device length (width), is displayed in Fig. 5(b). The value for the GFETs with h-BN is ~2250 cm^2^ V^−1^ s^−1^ at the carrier concentration of 1 × 10^12^ cm^−2^ in the hole-side, which is a factor 1.6 enhancement compared to that of the device without h-BN, ~1400 cm^2^ V^−1^ s^−1^. This enhancement is not very significant but still notable. A significant reduction of mobility would be expected if only the poor flatness of the h-BN was taken into account. This also implies that the high crystalline h-BN with much less charge impurities and higher optical phonon energies than SiO_2_ was produced.

In conclusion, pure borazine oligomer (B_3_N_3_H_4_)_x_ was synthesized by the polymerization reaction of borazine (B_3_N_3_H_6_) and used as a liquid precursor for the h-BN synthesis on nickel catalyst through the simple thermal annealing procedure. The nickel catalyst effectively promotes h-BN structure formation through the dehydro-crosslinking between borazine oligomers. Considering the Raman, FT-IR, XRD, XPS, TEM, SAED, UV-Vis and EELS characterization results, we can conclude the large areal h-BN film has been synthesized with high crystallinity. The electronic application of the h-BN film has been suggested by the enhanced characteristics of GFETs, showing the down-shift of Dirac point and the increase of the mobility. The coating method including the h-BN synthesis under Ni films and subsequent removal of Ni films has the potential for various functional applications such as insulations, thermal managements, and chemical or thermal protections.

## Methods

### Synthesis of the borazine oligomer

In order to synthesize borazine oligomer as precursor, borazine was prepared via dehydrogenation reaction of ammonia borane (BH_3_NH_3_) with nickel nanoparticles (NiNPs) at 80 °C. Ammonia borane is a reactant and nickel nanoparticles were used as catalyst in dehydrogenation reaction. The borazine (2.43 g, 30.1 mmol) was transferred into an evacuated Fischer-Porter glass pressure reaction vessel. The flask was allowed to warm to room temperature and then heated at 70 °C with the oil bath. The reaction was allowed to continue, with periodic degassing, until the liquid became sufficiently viscous for 48 h to remove the volatile materials at the room temperature at the nitrogen atmosphere for 48 h. Instead of removing with high vacuum system in the Sneddon’s paper^22^,^39^, leaving a white translucent gel (2.27 g, 93% yield based on reacting borazine). After completion of the reaction and removing the volatile materials, borazine oligomer (2.27 g) was dissolved in the chlorobenzene (24.49 g, 0.2 mol) with continuously stirring for 48 h while keeping the ratio of about 9 wt.%. And then the solution was filtrated with filter-paper at nitrogen atmosphere and room temperature for separating the practically insoluble materials. The filtrated solution was kept in a freezer at −45 °C for at least 3 weeks to have aging period in order to optimize its viscosity.

### Synthesis of the h-BN Films

Chlorobenzene solution of borazine oligomer was spin-coated onto an electrochemically polished nickel foil (99%, Nilaco Corp.) at 9000 rpm for 50 s. Since borazine oligomer is moisture sensitive, spin-coating was carried out inside a glove box that is continually purged with nitrogen. Then, the spin coated specimen was transported to the annealing reactor with container filled with nitrogen. The sample was then heated up to 1026 °C for 45 minutes under argon atmosphere and kept for 60 minutes for annealing. The reactor pressure was kept at 613 mtorr with the argon flow rate of 500 sccm for the annealing. After the synthesis, the chamber was slowly cooled down to room temperature under argon atmosphere. The h-BN film on nickel foil then was transferred to SiO_2_/Si or quartz substrate using graphene transfer process for characterization^40^.

### Synthesis of the h-BN Films under a Ni catalyst layer

In that method, borazine oligomer was spin-coated onto a silicon substrate with 1000 rpm inside a glove box, and a nickel catalyst layer was deposited onto the coated substrate by electron beam evaporator. The thickness of the film was about 300 nm. After deposition, annealing was followed at 1026 °C for 60 min under argon atmosphere, and nickel film was then etched by Iron (III) chloride (FeCl_3_). Thus, h-BN films were obtained directly on the silicon substrates.

### Device fabrication and analysis

The h-BN as the gate dielectric was transferred onto a 300 nm-thick SiO_2_/n+ Si substrate through a conventional transfer method using Polymethyl methacrylate (PMMA). In order to fabricate GFETs, the CVD-grown graphene films were formed onto a h-BN/SiO_2_/n+ Si substrate. Then, the channel region with a size of 3 μm × 3 μm was defined by photolithography and an inductively coupled plasma reactive ion etching system using O_2_ gas. As a final step, Cr (5 nm)/Au (50 nm) metals for the source/drain contacts were deposited using electron beam evaporator. Device performances were measured at room temperature under vacuum in a probe station system (Lakeshore 7500 series) using a Semiconductor Parameter Analyzer.

### Characterization

The transferred h-BN film was analyzed using Raman spectroscopy (Horiba, LabRAM HR, Ar laser, λ = 514 nm), Fourier transform infrared spectroscopy (FT-IR, Nicolet IS10), X-ray photoelectron spectroscopy (XPS, Thermo Scientific, K-ALPHA), X-ray diffraction (XRD, Rigaku, copper target), UV-Visible spectroscopy (UV-Vis, Jasco, V-670), and Transmission electron microscopy (TEM, Tecnai F20, 200 kV).

## Additional Information

**How to cite this article**: Park, S. *et al*. Facile Synthesis of Highly Crystalline and Large Areal Hexagonal Boron Nitride from Borazine Oligomers. *Sci. Rep.* **7**, 40260; doi: 10.1038/srep40260 (2017).

**Publisher's note:** Springer Nature remains neutral with regard to jurisdictional claims in published maps and institutional affiliations.

## Supplementary Material

Supplementary Information

## Figures and Tables

**Figure 1:**
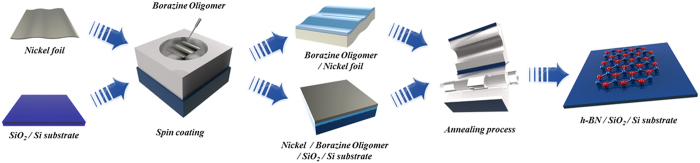
Schematic illustration of h-BN film formation from borazine oligomers.
