# Supplementary material for: Facile Synthesis of Highly Crystalline and Large Areal Hexagonal Boron Nitride from Borazine Oligomers
Source: Sci Rep. 2017 Jan 11;7:40260. doi: 10.1038/srep40260 (PMC5225468; doi:10.1038/srep40260)
Supplement: Supplementary Information [file srep40260-s1.pdf]

Facile Synthesis of Highly Crystalline and Large Areal Hexagonal Boron Nitride from Borazine Oligomers

Sungchan Park,[1] *Tae Hoon Seo,*[1] *Hyunjin Cho,*[1] *Kyung Hyun Min,*[1] *Dong Su Lee,*[1]

Dong-Il Won,[2] *Sang Ook Kang,*[2] *and Myung Jong Kim* [1,]*

[1] Applied Quantum Composites Research Center, Korea Institute of Science and Technology, Chudong-ro 92, Bongdong-eup, Wanju-gun, Jeollabuk-do, 565-905, Republic of Korea

[2] Department of Advanced Materials Chemistry, Korea University, 2511 Sejong-ro, Sejong, 30019 Republic of Korea

* **Corresponding author**. Tel.: +82-63-219-8135

E-mail: myung@kist.re.kr (M. J. Kim)

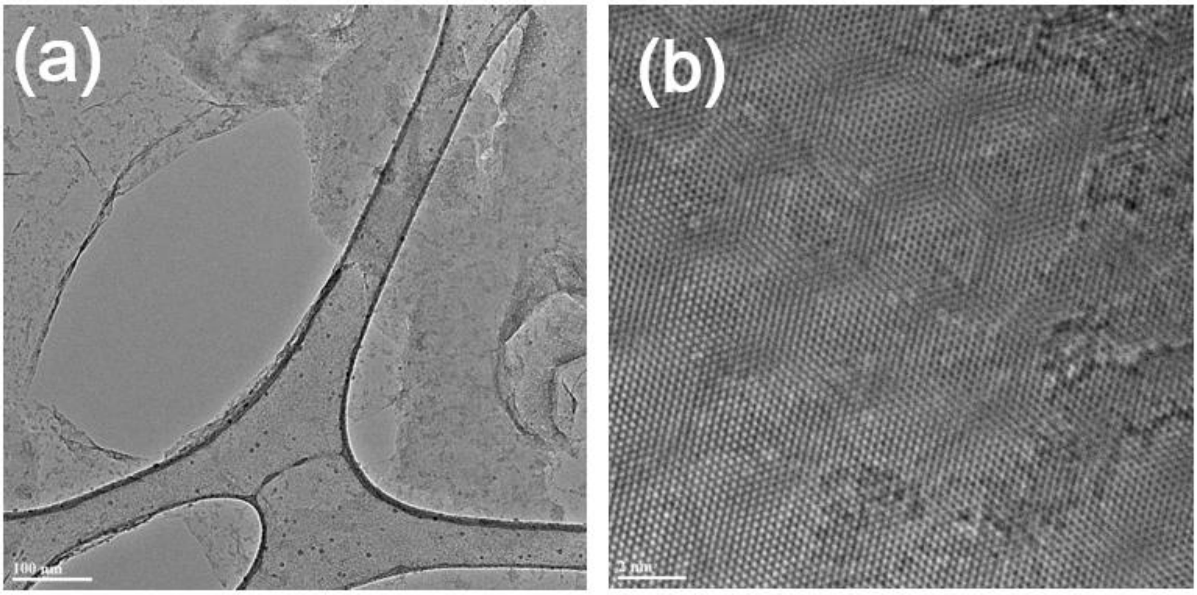

Figure S1. TEM image of the h-BN film. (a) Low-resolution and (b) high resolution TEM image.

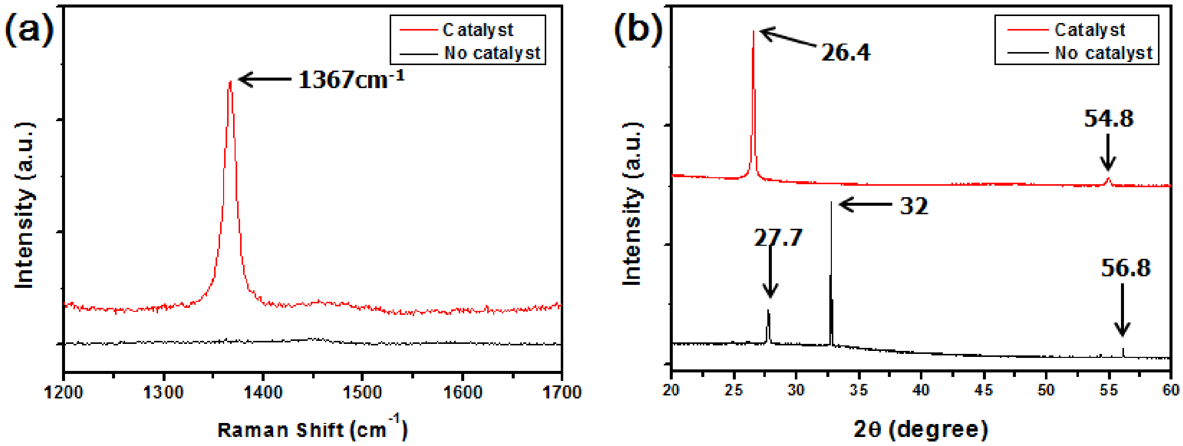

Figure S2. Raman (a) and XRD (b) data of h-BN films synthesized with and without Ni catalyst.

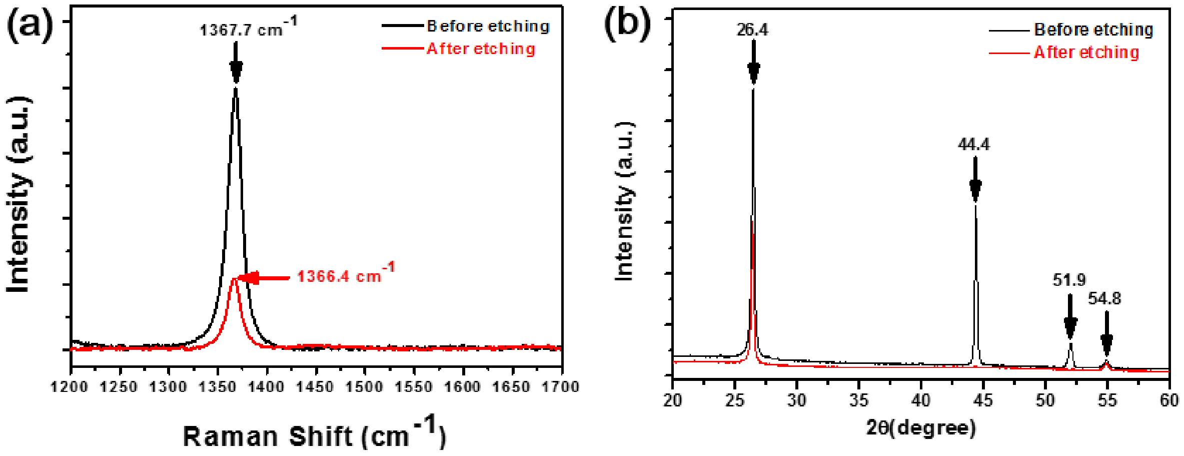

Figure S3. (a) Raman and (b) XRD data before and after removing a deposited catalyst layer.

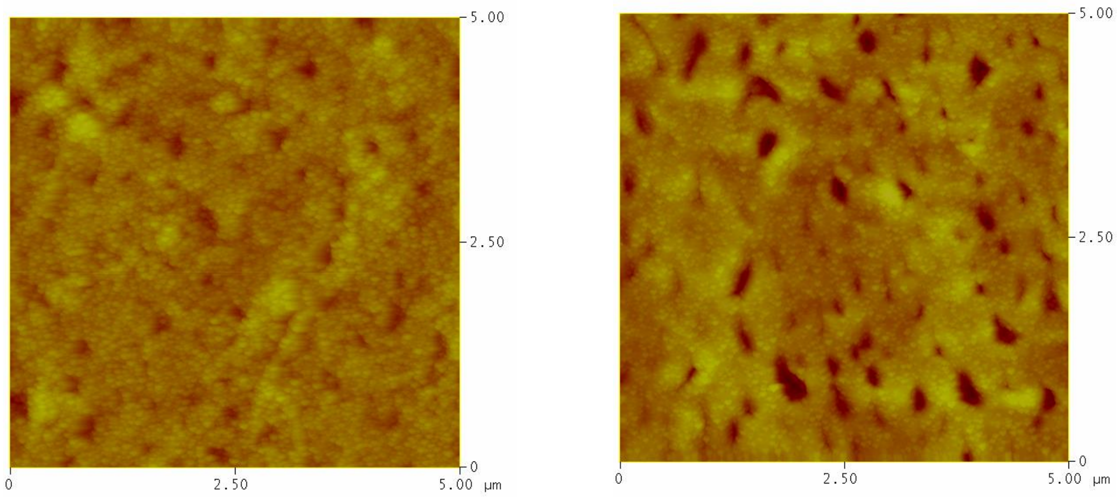

Figure S4. AFM images of the synthesized h-BN in different positions. The roughness was estimated to be ~ 6 and 9 nm in rms.

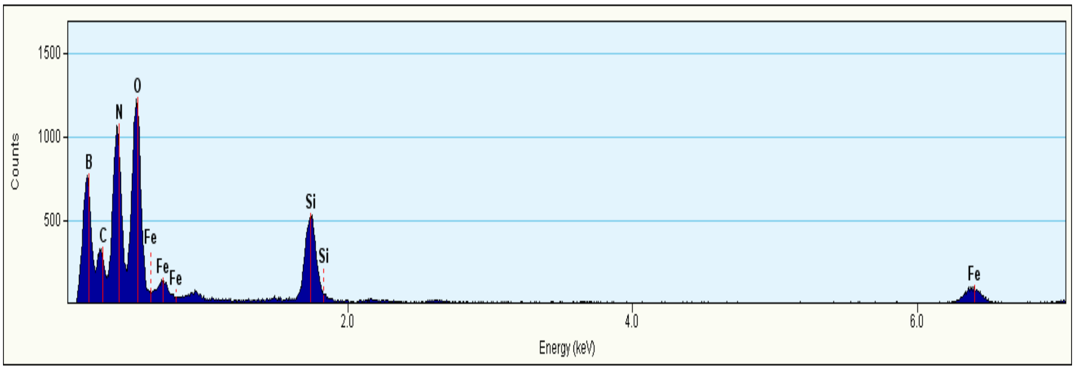

Figure S5. EDS (Energy Dispersive Spectroscopy) data in SEM equipment proved the existence of Fe atoms in the h-BN film, which was transferred on a SiO_2/Si substrate using $FeCl_3$ solution.
